# Clinical and imaging features predict mortality in COVID-19 infection in Iran

**DOI:** 10.1371/journal.pone.0239519

**Published:** 2020-09-24

**Authors:** Fatemeh Homayounieh, Eric W. Zhang, Rosa Babaei, Hadi Karimi Mobin, Maedeh Sharifian, Iman Mohseni, Anderson Kuo, Chiara Arru, Mannudeep K. Kalra, Subba R. Digumarthy

**Affiliations:** 1 Division of Thoracic Imaging and Intervention, Department of Radiology, Harvard University, Massachusetts General Hospital, Boston, Massachusetts, United States of America; 2 Department of Radiology, University of Medical Sciences, Firoozgar Hospital, Tehran, Iran; 3 Division of Cardiovascular Imaging, Department of Radiology, Harvard University, Massachusetts General Hospital, Boston, Massachusetts, United States of America; The Second Affilated Hospital, Zhejiang University School of Medicine, CHINA

## Abstract

The new coronavirus disease 2019 (COVID-19) pandemic has challenged many healthcare systems around the world. While most of the current understanding of the clinical features of COVID-19 is derived from Chinese studies, there is a relative paucity of reports from the remaining global health community. In this study, we analyze the clinical and radiologic factors that correlate with mortality odds in COVID-19 positive patients from a tertiary care center in Tehran, Iran. A retrospective cohort study of 90 patients with reverse transcriptase-polymerase chain reaction (RT-PCR) positive COVID-19 infection was conducted, analyzing demographics, co-morbidities, presenting symptoms, vital signs, laboratory values, chest radiograph findings, and chest CT features based on mortality. Chest radiograph was assessed using the Radiographic Assessment of Lung Edema (RALE) scoring system. Chest CTs were assessed according to the opacification pattern, distribution, and standardized severity score. Initial and follow-up Chest CTs were compared if available. Multiple logistic regression was used to generate a prediction model for mortality. The 90 patients included 59 men and 31 women (59.4 ± 16.6 years), including 21 deceased and 69 surviving patients. Among clinical features, advanced age (p = 0.02), low oxygenation saturation (p<0.001), leukocytosis (p = 0.02), low lymphocyte fraction (p = 0.03), and low platelet count (p = 0.048) were associated with increased mortality. High RALE score on initial chest radiograph (p = 0.002), presence of pleural effusions on initial CT chest (p = 0.005), development of pleural effusions on follow-up CT chest (p = 0.04), and worsening lung severity score on follow-up CT Chest (p = 0.03) were associated with mortality. A two-factor logistic model using patient age and oxygen saturation was created, which demonstrates 89% accuracy and area under the ROC curve of 0.86 (p<0.0001). Specific demographic, clinical, and imaging features are associated with increased mortality in COVID-19 infections. Attention to these features can help optimize patient management.

## Introduction

The rapid spread of the coronavirus disease 2019 (COVID-19) caused by the novel severe acute respiratory syndrome coronavirus 2 (SARS-CoV-2) has exerted unprecedented strain on the global healthcare system [1]. First described in case reports from Wuhan, China in December 2019, the novel coronavirus has since spread worldwide at an alarming pace [2, 3].

Due to the rapid dissemination and short period of awareness of the COVID-19 outbreak, the current understanding of the disease remains limited. Furthermore, most studies available on COVID-19 are currently based on data from China [4–6], with the limited reports available from less developed nations. Despite being one of the first countries affected by the COVID-19 outbreak, the clinical experience from Iran has been notably absent. The available literature suggests that COVID-19 infection is associated predominantly with fever, cough, and lymphocytopenia [6–8]. However, many subjects are either asymptomatic or do not manifest with fever or other respiratory symptoms [7]. It is unclear whether the presence of any specific symptom or laboratory anomaly carries particular significance. Similarly, with sporadic reports of young patients dying from COVID-19 [9], whether certain demographic groups demonstrate increased mortality from COVID-19 remains to be answered. Use of imaging in diagnosis and evaluation of suspected or known COVID-19 infection is variable among different countries. Given its low specificity and overall predictive value [10], imaging features are currently not considered helpful for diagnosis of COVID-19 infection by most clinicians. The American College of Radiology and the Society of Thoracic Radiology do not recommend chest computed tomography (CT) for screening or diagnosis of COVID-19 [11]. These recommendations are echoed by the World Health Organization (WHO) consensus guidelines, which recommend the use of reverse-transcription polymerase chain reaction (RT-PCR) over chest imaging for the diagnosis of COVID-19 [12]. Local Iranian practices reiterate this sentiment, advocating the use of repeat chest CT in high-risk hospitalized patients to assess treatment response and to address clinical conundrums [13]. When obtained, common findings on chest CT range from normal to peripheral ground-glass opacities to more diffuse parenchymal opacities [14].

In this study, we analyze the clinical and radiologic factors that correlate with mortality odds in COVID-19 positive patients from a tertiary care center in Tehran, Iran. A prediction model was attempted using the patient-specific data.

## Materials and methods

The study was approved by the Partners institutional review board for retrospective analyses of the data with permission from the responsible personnel at the local hospital. Between February 10, 2020 and March 30, 2020, 90 consecutive hospitalized patients with RT-PCR confirmed COVID-19 infection were included in this study from a tertiary hospital (Firoozgar Hospital, Tehran, Iran). The RT-PCR tests were performed on either throat or nasal swabs, or both. All patients underwent CT scanning of the chest. A subset of patients also had chest radiographs acquired at the time of admission. The patient demographics, symptoms at presentation, vitals, laboratory values, and hospital course were extracted from medical records. Survival was the outcome of interest.

### Clinical and laboratory data

The extracted clinical data included the nature of symptoms, duration of symptoms before hospital visit, the presence of other pre-existing medical conditions (including asthma, chronic obstructive pulmonary disease, diabetes, hypertension, ischemic heart disease, cerebrovascular disease, malignancy, chronic renal disease, immunodeficiency, and autoimmune conditions), and results of laboratory analyses including total white blood cell (WBC) count, absolute lymphocyte count, percentage of lymphocytes, C-reactive protein (CRP) level, lactate dehydrogenase (LDH) level, and erythrocyte sedimentation rate (ESR). Body temperature and oxygen saturation at presentation were also recorded.

### Imaging technique and evaluation

CT images were acquired using a 6-slice multi-detector scanner (SOMATOM Emotion, Siemens Healthineers, Erlangen, Germany) with 110–130 kV, 80 mAs using automatic exposure control technique (Care Dose 4D), gantry rotation time of 0.8 second, pitch of 1.35:1, and slice thickness of 2–2.5 mm. The images were reconstructed with standard soft tissue and high-resolution lung kernels. All the examinations were obtained without the administration of IV contrast. Twenty -five patients had at least one follow-up chest CT done with the same protocol. Portable or upright radiographs were performed and were available for review in 35 patients. The CT images and radiographs were anonymized then reviewed on a high-resolution monitor using RadiAnt DICOM Viewer v5.5.1 (Medixant, Poland). All CT and radiograph images were reviewed independently by two fellowship-trained board-certified thoracic radiologists with at least 14 years of experience (SRD and MK) by consensus.

### CT image analysis

**Fig 1:**
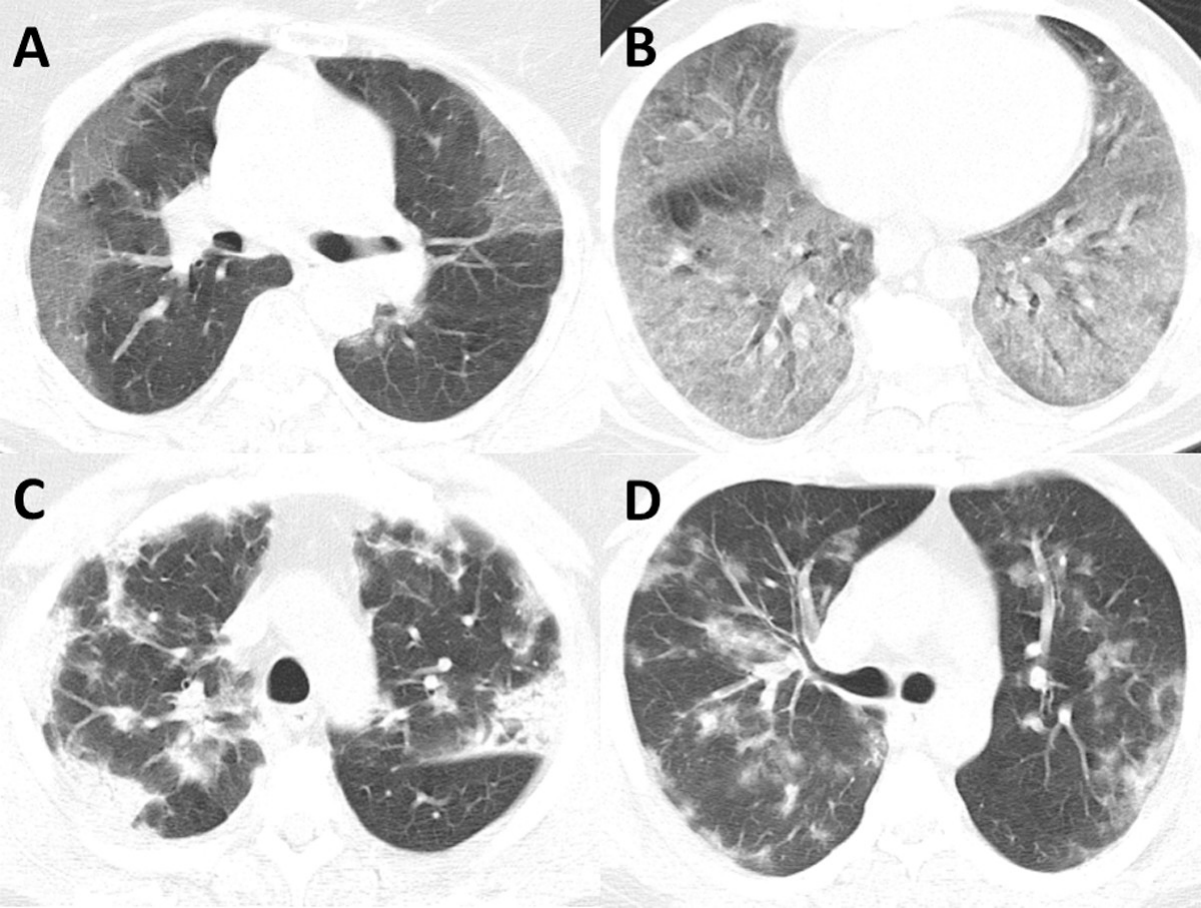
Examples of opacification patterns of COVID-19 on CT imaging. (A) Most common presentation of COVID-19 on CT imaging with multifocal peripheral ground-glass opacities. (B) Example of diffuse multi-lobar ground-glass opacities in COVID-19. (C) Example of consolidative and ground-glass opacities with both peripheral and central distribution. (D) Example of rare bilateral nodular consolidations seen in COVID-19.

The image analysis was performed on mediastinal window setting for assessment of mediastinal nodes, the diameter of the main pulmonary artery (MPA), and pleural effusions. The MPA was measured in the axial plane perpendicular to the vascular axis just above the level of bifurcation. A lymph node measuring 10 mm or larger in the short axis was considered positive. The assessment of lung parenchyma was performed on high-resolution lung window settings. The pattern of lung opacity in each lobe (Fig 1) was classified as (a) pure ground-glass, (b) ground-glass with areas of consolidation, (c) reverse halo, (d) nodular, or (e) mixed. The extent of the parenchymal opacity in each lobe was graded on a 6-point numeric scale (0: none, 1: minimal <5%, 2: mild 5–25%, 3: moderate 25–50%, 4: moderate-severe 51–75%, and 5: severe >75%), as described previously by Pan et al. [15]. Based on the numerical score of extent in each lobe and the number of lobes affected, a CT severity score was then calculated by summing the numeric value assigned to each lobe (values ranging from 0 to 25). The lungs were also assessed for bronchiectasis and stigmata of prior granulomatous disease/tuberculosis.

### Chest radiograph analysis

**Fig 2:**
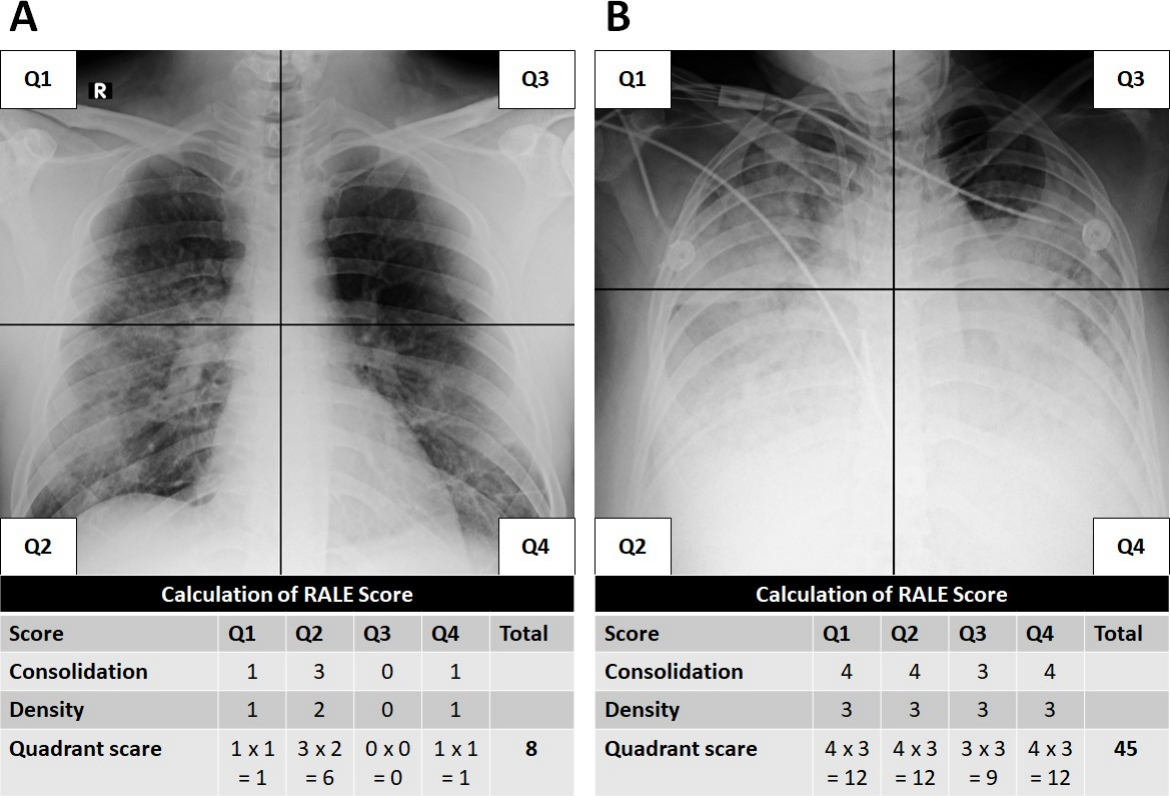
Examples of RALE scoring in COVID-19 positive patients. (A) Initial frontal chest radiograph in a 32-year-old male from the survivor cohort with total RALE score of 8. (B) Initial frontal chest radiograph in a 48-year-old female from the deceased cohort with total RALE score of 45.

Chest radiographs were obtained as clinically indicated using portable x-ray units. All chest radiographs were acquired using single frontal technique with anteroposterior projection in either supine or sitting position. The assessment of chest radiographs was done using the Radiographic Assessment of Lung Edema (RALE) score, originally described to standardize the description of diffuse lung opacities for acute respiratory distress syndrome (ARDS) [16]. In this method, a radiograph is divided into four quadrants by drawing a horizontal line by the first branch of the left main bronchus and vertical line through the mid vertebral bodies (Fig 2). Each quadrant is assigned a consolidation score of 0–4 based on the extent of pulmonary opacities (0: none, 1: minimal <25%, 2: mild 25–50%, 3: moderate 50–75%, 4: severe >75%) and a density score of 1–3 based on the density of opacities (1: hazy, 2: moderate, 3: dense). A RALE score is obtained in each quadrant by multiplying the consolidation score and the density score, yielding the quadrant score (0 to 12). The final RALE score is the sum of quadrant scores (0 to 48).

### Statistical analysis

The clinical and imaging characteristics between the deceased patients and survivors were compared. Two-tailed unpaired Student’s t-test with Welch’s correction was used for continuous data. Fisher’s exact test was used for binary categorical variables. Pearson’s chi-square test was used for nonbinary nominal categorical variables. Cochran-Armitage chi-square test for trend was used for ordinal categorical variables. The specific tests used are indicated in table legends. Given the small dataset relative to the number of parameters examined, there was no correction for multiple comparisons, acknowledging this decision impacts the family-wise error rate.

Using multiple logistic regression, modeling was then attempted. The stepwise forward selection was used with a likelihood ratio test for between-model comparison. The area under the receiver operating characteristic curve (AUC) was calculated along with positive predictive value, negative predictive value, and overall accuracy. Significance was deemed at p < 0.05 for all tests. Statistical analysis was performed using GraphPad Prism 8.3.0 (San Diego, California).

## Results

### Demographics, symptoms and pre-existing medical conditions

Among the 90 patients, there were 21 deceased patients (23%) and 69 survivors (77%). Group-specific patient details, duration of symptoms before the initial visit, duration of hospital stay, and co-morbidities are summarized in Table 1. The deceased patients were overall older (p = 0.02). The time from symptoms onset to the presentation was significantly shorter in the deceased group compared to survivors (p = 0.01), as were the number of days in ICU stay (p = 0.003). Number of days of hospital admission was not a significant predictor of mortality (p = 0.08).

**Table 1:** Demographics, co-morbidities, and duration of symptoms and hospitalization.

	Deceased (n = 21)	n	Survivor (n = 69)	n	p value
**Sex**					
Male	15 (71.4%)	21	43 (62.3%)	69	0.6
**Age**					
Age (years)*	68.24 ± 19.21	21	56.94 ± 15.00	69	0.02
**Durations**					
Symptoms to admission*	3.4 ± 3.8	12	6.9 ± 3.9	60	0.01
Number of days admitted*	12.6 ± 11.3	21	8.0 ± 3.6	65	0.08
Number of days in ICU*	9.1 ± 11.2	21	0.7 ± 2.5	65	0.003
**Co-morbidities**					
COPD and asthma	2 (13%)	15	4 (7%)	60	0.6
Diabetes	5 (33%)	15	20 (33%)	60	>0.9
Hypertension	5 (33%)	15	24 (40%)	60	0.8
Ischemic heart disease	5 (33%)	15	13 (22%)	60	0.3
Cerebrovascular disease	0 (0%)	15	1 (2%)	60	>0.9
Malignancy	1 (7%)	15	0 (0%)	60	0.2
Chronic renal disease	0 (0%)	15	2 (3%)	60	>0.9
Immunodeficiency	2 (13%)	15	3 (5%)	60	0.3
Autoimmune disease	1 (7%)	15	0 (0%)	60	0.2

*Two tailed unpaired Student’s t test with Welch correction. Otherwise, Fischer exact test.

Data regarding symptoms and mortality are shown in Table 2. Loss of consciousness was seen only in the deceased group (28%, 5/21). Myalgia was more common among the survivors than the deceased (42% vs. 11%). Sore throat was reported only in the deceased group (11%, 2/21). The survivors reported more symptoms in general compared to the deceased (p = 0.03). The presence of a pre-existing medical condition was not statistically different between the groups for any condition examined. The number of pre-existing medical conditions was not different between groups (p = 0.3).

**Table 2:** Symptoms at presentation.

	Deceased (n = 21)	n	Survivor (n = 69)	n	p value*
Symptoms*					
Fever	10 (56%)	18	45 (73%)	62	0.2
Chills	2 (11%)	18	18 (29%)	62	0.2
Fatigue	2(11%)	18	8 (13%)	62	>0.9
Myalgia	2 (11%)	18	26 (42%)	62	0.02 Odds ratio: 0.17 (0.04–0.77)
Chest pain	2 (11%)	18	2 (3%)	62	0.2
Shortness of breath	13 (72%)	18	37 (60%)	62	0.4
Cough	8 (44%)	18	42 (67%)	62	0.1
Headache	0 (0%)	18	6 (10%)	62	0.3
Sore throat	2 (11%)	18	0 (0%)	62	0.048 Odds ratio: inf (1.65-inf)
Nonpsychiatric anorexia	0 (0%)	18	1 (2%)	62	>0.9
Nausea and vomiting	0 (0%)	18	9 (15%)	62	0.2
Diarrhea	0 (0%)	18	3 (5%)	62	>0.9
Sputum production	0 (0%)	18	2 (3%)	62	>0.9
Loss of consciousness	5 (28%)	18	0 (0%)	62	0.0004 Odds ratio: inf (5.85-inf)
Hemoptysis	0 (0%)	18	0 (0%)	62	>0.9
Number of symptoms^#^	1: 5 (28%) 2: 2 (11%) 3: 7 (39%) 4: 4 (22%) 5: 0 (0%) 6: 0 (0%)	18	1: 3 (5%) 2: 12 (19%) 3: 26 (42%) 4: 12 (19%) 5: 8 (13%) 6: 1 (2%)	62	0.03

*Fischer exact test.

^#^Chi-square (Cochran-Armitage) test for trend.

### Laboratory values and vital signs

The laboratory data and vital signs are summarized in Table 3. The oxygen saturation was lower among the deceased (p < 0.001). The WBC count was higher in the deceased group (p = 0.02), but the lymphocyte fraction is lower (p = 0.03). The resultant absolute lymphocyte count is borderline higher in the survivor group (p = 0.05). The platelet count was lower in the deceased cohort (p = 0.048) but was affected by a single patient with deficient platelets from idiopathic thrombocytopenia. After the removal of this outlier, the difference was no longer significant. There was no difference in ESR, CRP, or LDH between groups, although we note significant portions of the data were missing for these values.

**Table 3:** Temperature, oxygen saturation, and blood tests during admission.

	Deceased	n	Survivor	n	p value*
Temperature (°C)	37.5 ± 0.8	15	37.5 ± 0.7	62	>0.9
pO_2_ (%)	84.7 ± 9.6	15	92.2 ± 4.3	60	<0.001
WBC (per ml)	8429 ± 4631	21	5851 ± 1855	69	0.02
Lymphocytes (per ml)	953 ± 439	21	1173 ± 439	69	0.05
Lymphocyte fraction (%)	14.9 ± 12.5	21	21.6 ± 8.8	69	0.03
Platelets (per ml)^#^	155857 ± 56971	21	185304 ± 59704	69	0.048
ESR (mm/hour)	40.9 ± 25.0	17	45.62 ± 22.4	61	0.5
CRP (mg/L)	55.2 ± 49.4	11	48.3 ± 42.3	26	0.7
LDH (IU/L)	607.6 ± 211.4	15	643.4 ± 329.4	49	0.6

*Two tailed unpaired Student’s t test with Welch correction.

^#^Difference no longer significant after removing of one outlier in the deceased group.

### Imaging analysis

Imaging findings on the initial CT of the chest are summarized in Table 4 and follow-up CT changes in Table 5. Pleural effusions on initial CT were more common in the deceased than survivors (p = 0.005). There was no detected difference in pulmonary artery diameter, mediastinal/hilar lymphadenopathy, bronchiectasis or prior tuberculosis. The right middle lobe (p = 0.04) and left lower lobe (p = 0.02) were more severely affected in deceased patients than survivors. The combined lung severity score was not significant (p = 0.08) if all five lobes were included and significant (p = 0.04) if only the middle and lower lobes were included. The number of lobes involved was not different between groups (p = 0.3). While the pattern of lung opacification was not different between groups, we note that pure ground-glass opacities were most common (>60% for all lobes). Geographic distribution of disease demonstrated a trend toward higher diffuse distribution in the deceased group but did not reach significance (p = 0.09).

**Table 4:** Chest CT findings on initial CT examination.

	Deceased (n = 21)	Survivor (n = 69)	p value
**Non-parenchymal Findings**			
Pulmonary artery diameter*	2.8 ± 0.4	2.6 ± 0.3	0.1
Pleural effusion	10 (48%)	10 (14%)	0.005
Prior tuberculosis	3 (14%)	8 (12%)	0.7
Mediastinal or hilar lymphadenopathy	11 (52%)	24 (35%)	0.2
Bronchiectasis	4 (19%)	6 (9%)	0.2
**Lung Parenchyma**			
Right upper lobe severity^#^	0: 1 (5%) 1: 4 (19%) 2: 4 (19%) 3: 6 (29%) 4: 2 (10%) 5: 1 (19%)	0: 3 (4%) 1: 13 (19%) 2: 26 (38%) 3: 13 (19%) 4: 12 (17%) 5: 2 (3%)	0.2
Right middle lobe severity^#^	0: 2 (10%) 1: 4 (19%) 2: 5 (24%) 3: 5 (24%) 4: 0 (0%) 5: 5 (24%)	0: 12 (17%) 1: 20 (29%) 2: 15 (22%) 3: 16 (23%) 4: 2 (3%) 5: 4 (6%)	0.04
Right lower lobe severity^#^	0: 0 (0%) 1: 2 (10%) 2: 3 (14%) 3: 8 (38%) 4: 3 (14%) 5: 5 (24%)	0: 2 (3%) 1: 6 (9%) 2: 21 (30%) 3: 22 (32%) 4: 12 (17%) 5: 6 (9%)	0.1
Left upper lobe severity^#^	0: 0 (0%) 1: 6 (29%) 2: 4 (19%) 3: 6 (29%) 4: 2 (10%) 5: 3 (14%)	0: 6 (9%) 1: 11 (16%) 2: 24 (35%) 3: 15 (22%) 4: 12 (17%) 5: 1 (1%)	0.3
Left lower lobe severity^#^	0: 1 (4%) 1: 2 (10%) 2: 2 (10%) 3: 6 (29%) 4: 4 (19%) 5: 6 (29%)	0: 4 (6%) 1: 6 (9%) 2: 28 (41%) 3: 12 (17%) 4: 15 (22%) 5: 4 (5%)	0.02
Total lung severity score*	14.6 ± 6.4	11.8 ± 4.9	0.08
Lower lung zone severity score*	9.2 ± 3.9	7.2 ± 3.3	0.04
Number of lobes involved	2: 0 (0%) 3: 1 (5%) 4: 2 (10%) 5: 18 (86%)	2: 2 (3%) 3: 6 (9%) 4: 9 (13%) 5: 52 (75%)	0.3
**Opacification pattern**^$^			
Right upper lobe opacification pattern^a^	0: 1 (5%) 1: 13 (62%) 2: 5 (24%) 3: 0 (0%) 4: 1 (5%) 5: 1 (5%)	0: 3 (4%) 1: 51 (74%) 2: 13 (19%) 3: 1 (1%) 4: 1 (1%) 5: 0 (0%)	0.4
Right middle lobe opacification pattern^a^	0: 2 (10%) 1: 13 (62%) 2: 4 (19%) 3: 0 (0%) 4: 1 (5%) 5: 1 (5%)	0: 12 (17%) 1: 46 (67%) 2: 9 (13%) 3: 1 (1%) 4: 1 (1%) 5: 0 (0%)	0.4
Right lower lobe opacification pattern^a^	0: 0 (0%) 1: 14 (67%) 2: 5 (24%) 3: 0 (0%) 4: 1 (5%) 5: 1 (5%)	0: 2 (3%) 1: 49 (71%) 2: 17 (25%) 3: 1 (1%) 4: 0 (0%) 5: 0 (0%)	0.2
Left upper lobe opacification pattern^a^	0: 0 (0%) 1: 13 (62%) 2: 6 (29%) 3: 0 (0%) 4: 1 (5%) 5: 1 (5%)	0: 6 (9%) 1: 48 (70%) 2: 14 (20%) 3: 1 (1%) 4: 0 (0%) 5: 0 (0%)	0.1
Left lower lobe opacification pattern^a^	0: 0 (0%) 1: 13 (62%) 2: 6 (29%) 3: 0 (0%) 4: 1 (5%) 5: 1 (5%)	0: 4 (6%) 1: 48 (70%) 2: 16 (23%) 3: 1 (1%) 4: 0 (0%) 5: 0 (0%)	0.1
Distribution of disease^b^	1: 11 (52%) 2: 6 (29%) 3: 4 (19%)	1: 51 (74%) 2: 14 (20%) 3: 4 (6%)	0.09

*Two tailed unpaired Student’s t test with Welch correction.

^#^Chi-square (Cochran-Armitage) test for trend.

^$^Pearson’s chi-square test. Otherwise, Fischer’s exact test.

^a^0: no involvement, 1: pure ground-glass, 2: ground-glass with consolidation, 3: reverse halo, 4: nodular, 5: mixed.

^b^1: subpleural/peripheral, 2: subpleural and central distribution, 3: diffuse distribution.

**Table 5:** Change in imaging findings between scans.

	Deceased (n = 6)	Survivor (n = 19)	p value
Subgroup age (years)*	67.7 ± 16.5	57.3 ± 14.0	0.2
Subgroup sex (male)	4 (67%)	14 (74%)	>0.9
Time between scans (days)*	5.3 ± 1.2	5.1 ± 2.2	0.7
**Nonparenchymal Findings**			
Change in pulmonary artery diameter*	0.42 ± 0.52	0.02 ± 0.33	0.1
Development of pleural effusion	2/4	0/15	0.04
Development of mediastinal or hilar lymphadenopathy	4/5	4/15	0.1
Development of bronchiectasis	2/4	2/19	0.1
**Lung Parenchyma**			
Change in total lung severity score*	10.2 ± 5.5	3.1 ± 7.9	0.03
Total lung severity score on CT*	20.2 +/- 2.7	14.1 +/- 6.8	0.004
Lower lung zone severity score*	12.7 ± 2.5	9.0 ± 3.8	0.02

*Two tailed unpaired Student’s t test with Welch correction. Otherwise, Fischer’s exact test.

**Fig 3:**
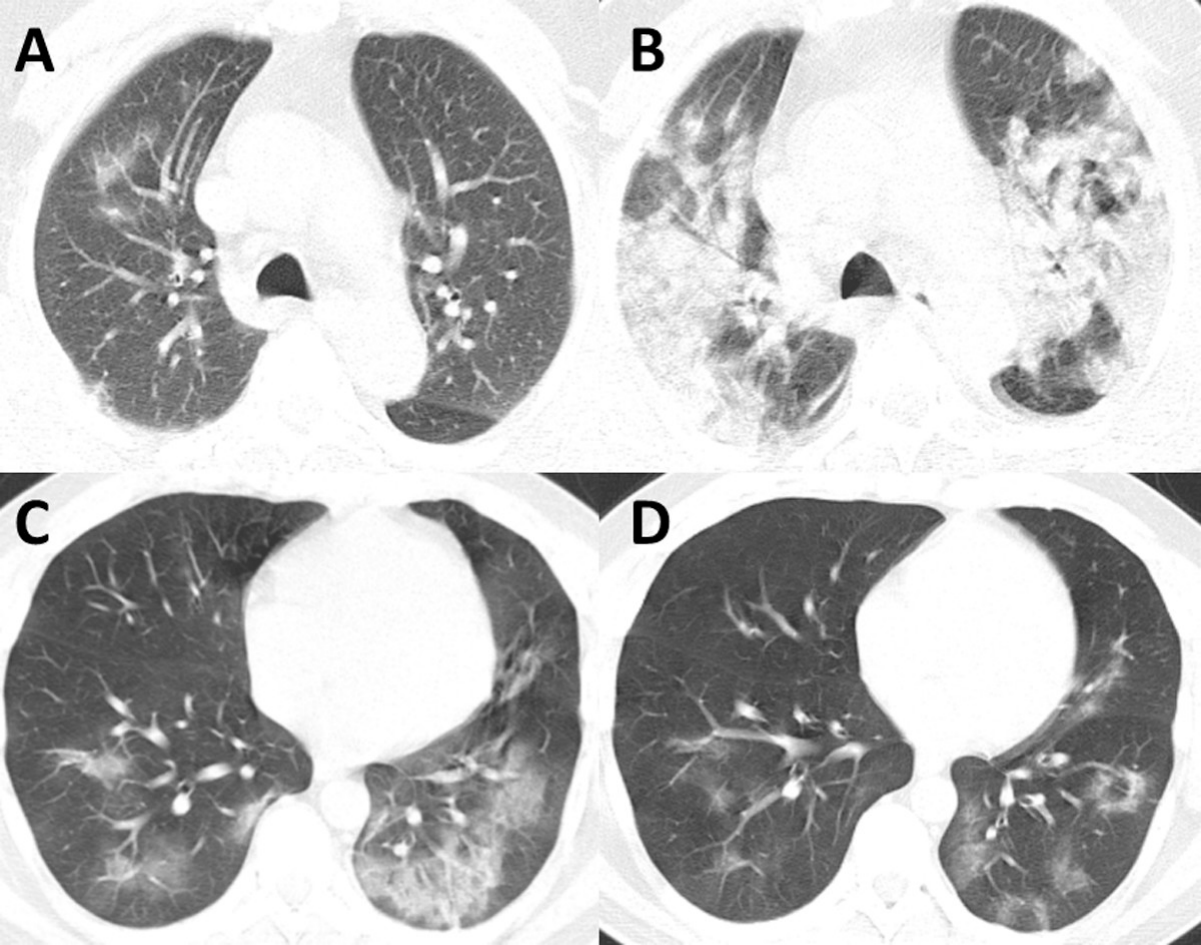
Two cases from the survivor and deceased cohorts illustrating progression of CT findings over time in COVID-19. (A) Admission CT images of a 69-year-old male from the deceased cohort demonstrating mild bilateral peripheral ground-glass opacities on CT. Total CT severity score calculated at 9. (B) Follow-up CT images of the same patient 6 days later with severe worsening of disease manifesting as diffuse consolidative and ground-glass opacities. Total CT severity score calculated at 22. (C) Admission CT images of a 37-year-old male from the survivor cohort demonstrating multilobar ground-glass opacities. Total CT severity score calculated at 14. (D) Follow-up CT images of the same patient 6 days later with significant improvement of disease extent. Total Ct severity score calculated at 10.

On follow-up CT, combined lung severity score, either of all lobes (p = 0.004) or of only middle and lower lobes (p = 0.02), was significant between the deceased and survivors (Fig 3). Interval development of pleural effusion was seen only in the deceased group (p = 0.04). The remaining characteristics were not different. The time-interval between two CT exams was similar between deceased patients and survivors (p = 0.7).

The results regarding the RALE scores are shown in Table 6. The RALE scores were higher in the deceased patients in sum (p = 0.002) as well as in individual quadrants (p = 0.002–0.03).

**Table 6:** Chest radiograph RALE scores.

	Deceased (n = 14)	Survivor (n = 21)	p value
Total score	28.9 ± 13.3	14.5 ± 10.2	0.002
Right upper quadrant score	6.3 ± 3.8	3.4 ± 3.4	0.03
Right lower quadrant score	8.1 ± 3.3	5.0 ± 3.6	0.02
Left upper quadrant score	5.4 ± 4.5	1.9 ± 2.9	0.02
Left lower quadrant score	9.1 ± 4.2	4.4 ± 3.4	0.002

* Two tailed unpaired Student’s t test with Welch correction.

**Fig 4:**
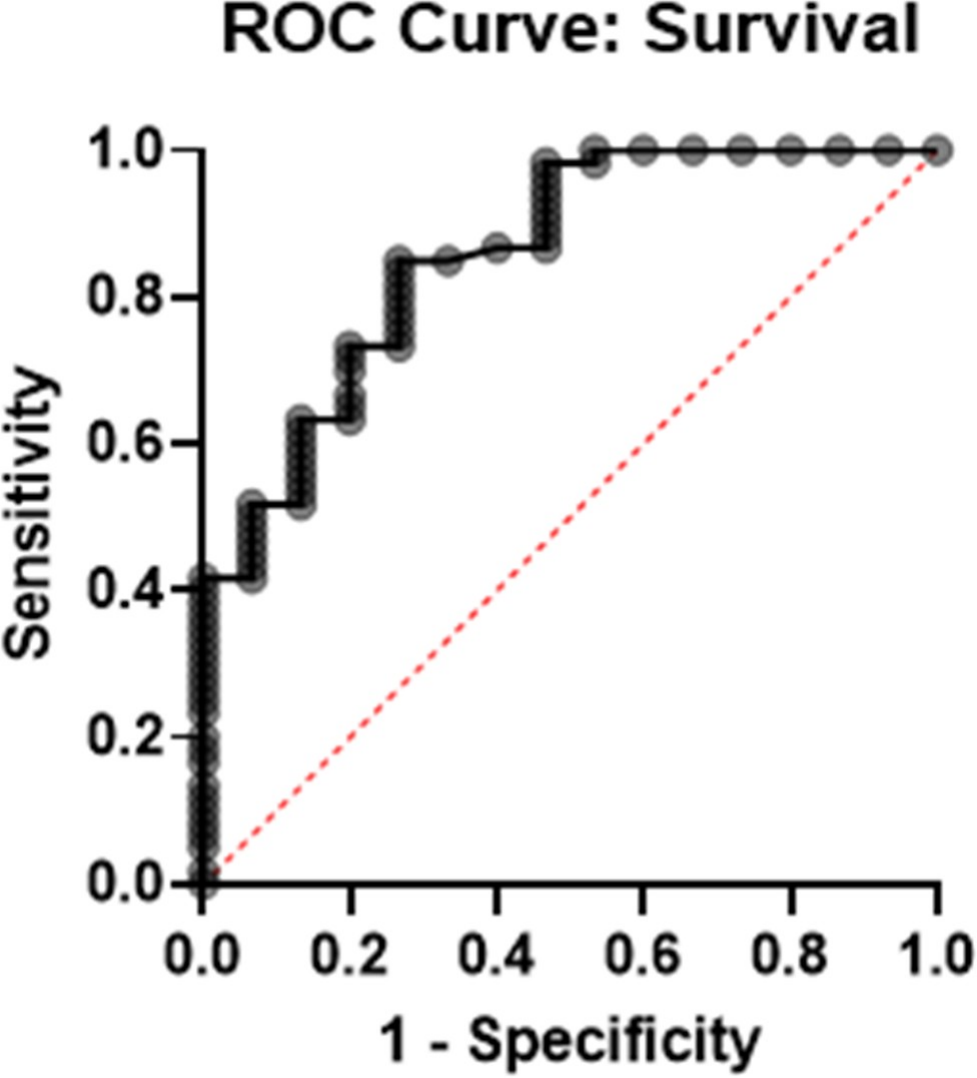
ROC curve of the two-factor logistic regression model in predicting survival in COVID-19 infection using age and oxygen saturation on presentation. AUC = 0.86.

Results regarding the logistic regression model are shown in Table 7 with the ROC curve in Fig 4. The stepwise selection terminated at two predictors: age and oxygen saturation. The model suggested age (in year, odds ratio: 0.92, p = 0.004) and oxygen saturation (in percent, odds ratio: 1.21, p = 0.002) are independently predictive of survival. The AUC was estimated at 0.86. Using the current dataset, the positive predictive value, negative predictive value, and overall accuracy were calculated at 89%, 89%, and 89%, respectively.

**Table 7:** Two-factor logistic regression model for survival prediction.

Variable	β	SE	OR	95% CI for OR	p value
Age (years)	-0.082	0.029	0.921	0.863–0.979	0.004
pO2 (%)	0.188	0.061	1.207	1.085–1.390	0.002
Intercept	-9.902	5.181	-	-	0.056
**ROC**	AUC	SE	95% CI	p value	
	0.857	0.05	0.752–0.963	<0.0001	
Characteristics*	Sensitivity	Specificity	Positive PV	Negative PV	Accuracy
	98%	53%	89%	89%	89%
**Log-likelihood ratio test**	Statistic	p value			
	25.71	<0.0001			

Abbreviations: CI, confidence interval; OR, odds ratio; SE, standard error; ROC, receiver operating curve. *Model characters in predicting survival in obtained dataset, not validated with testing dataset.

## Discussion

In this study, we found younger age, higher oxygen saturation, lower WBC count, increased lymphocyte fraction, presence of myalgia, lack of sore throat, lack of loss of consciousness, a higher number of symptoms, lower RALE score, lower CT severity score on follow-up CT, and absence of pleural effusion correlated with survival. In particular, age and oxygen saturation were independent markers of survival that may be used to generate a prediction model. Overall, our findings suggest that there are demographic, clinical, and radiologic features that are associated with mortality in COVID-19 infections that warrant attention in patient management.

In our study, the time from symptom onset to presentation is longer in the survivors compared to the deceased group. This difference may imply a more rapid course of disease and deterioration. These findings are in line with previously published data from the Chinese Center for Disease Control, outlining differences in clinical severity in COVID-19 ranging from mild disease to critical forms that involve rapid evolution of lung infiltrates > 50% within 24 to 48 hours and multiorgan failure [17]. The factors behind this rapid deterioration are not well understood at this time. However, there is growing evidence suggesting that severe forms of COVID-19 may be related to inflammatory dysregulation and cytokine storm syndrome [18].

Our study reiterates older age as a risk factor for poor prognosis in COVID-19, consistent with findings from previous reports [7, 17, 19]. Older age was also reported to be associated with adverse clinical outcomes in patients with SARS and MERS [20, 21]. Based on a prior animal study, older individuals may have an increased and prolonged host immune response to SARS-CoV infection, which may underlie the poorer outcomes [22, 23].

In terms of vital signs and laboratory parameters, the lower oxygen saturation (pO2) and lower lymphocyte fraction in the deceased group echo findings from prior studies [24, 25]. Likewise, the WBC count is significantly higher in the deceased group, similar to a cohort study from China [25]. The reason for the lymphocyte deficiency is uncertain but may be related to the increased propensity of the SARS-CoV-2 virus to infect lymphocytes, cytokine-mediated apoptosis of lymphocytes, or inhibition of lymphocyte production [8, 26]. Unlike prior studies [27, 28], inflammatory markers, including CRP, ESR, and LDH, were not statistically different in this study. However, not all patients were tested for these inflammatory markers, and thus their role may be underestimated.

Consistent with prior studies [25, 29], presenting clinical symptoms alone were rarely good predictors of outcome. The loss of consciousness was seen in a quarter of the deceased group, but not in the survivors. It is unclear whether the reported loss of consciousness resulted from syncopal, cardiopulmonary, epileptic, frailty, psychogenic, or other underlying causes. The higher prevalence of myalgia, as well as the higher overall number of symptoms reported in the survivors, may originate from more robust activation of a subset of the immune response [28], longer duration allowed for symptom emergence, or possibly artifactual from reporting bias in the sicker patients.

In keeping with the results from prior studies, our findings highlight the high prevalence of comorbidities in hospitalized patients with COVID-19, with more than half of patients affected by at least one comorbidity [19, 30, 31]. The three most common comorbidities are hypertension, diabetes, and ischemic heart disease, reflecting the high prevalence of these conditions in general and consistent with literature [19, 30, 31]. Furthermore, similar to reports from China, the frequency of COVID-19 patients with comorbid respiratory diseases, chronic renal disease, and malignancy is relatively low [29]. The reasons for this observation are speculative but may relate to differences in healthcare systems and screening in the community [29]. Interestingly, there was no significant difference in comorbidities between the survivor and deceased groups. Mortality rate of 23% in our subjects was also significantly higher than the data from China and elsewhere, where the mortality rate between 5.6 to 15.2% was reported [32]. However, multiple subsequent studies have demonstrated poor outcomes in COVID-19 patients with comorbidities, particular in those with cardiovascular diseases and diabetes [33, 34]. We believe this difference is likely attributed to selection bias. Given the population that we studied had multiple comorbidities at baseline, they were also likely to have more comorbidities regardless of outcome. There was likely an under-representation of healthier patients without comorbidities who did not require hospitalization.

Our chest CT imaging findings of predominantly multilobar, bilateral groundglass pulmonary opacities are consistent with features reported by previous meta-analyses [35, 36]. The association between the presence of pleural effusions, an uncommon finding on imaging for COVID-19, and mortality may indicate a more severe disease variant or relate to underlying cardiovascular disease, either of which may explain the poor prognosis. The development of pleural effusion is likely a related finding. The total lung CT severity score approached but did not reach statistical significance on the initial chest CTs between the alive and deceased cohorts. On follow-up CT imaging, higher/progression of CT severity score in the deceased group implicates once again a more rapid disease deterioration.

A higher RALE score on initial chest radiographs correlated with patient mortality. Previous studies on RALE scoring in ARDS demonstrated high inter-rater agreement [16, 37], suggesting it may serve as a standardized tool for the initial assessment of COVID-19. Given that role for CT imaging in COVID-19 appears limited in terms of accuracy and predictive values [10, 11], RALE scoring on chest radiographs could serve as a more convenient and easily implementable method for assessing and triaging patients diagnosed with COVID-19.

We acknowledge a few limitations. First, selection bias is likely present, favoring sicker patients with more comorbidities that were hospitalized for COVID-19. Due to the retrospective study design, the clinical and radiologic findings were not obtained in all the patients and there was a relatively small sample size, limiting the detection of differences in some clinical and radiologic features. For example, we were unable to obtain consistent data on arterial blood gases, an important component of hypoxemia assessment that will need to be examined in subsequent studies. We note that we did not correct for multiple comparisons in this pilot study due to limitations on sample size, acknowledging the resultant increase in family-wise error rate. Lastly, extrapolation of our findings should be done with caution, given the differences in socioeconomic status and health care systems between Iran and the other countries. Future studies with larger sample size and power, particularly in developing countries, may help validate our results.

In summary, our study demonstrated several clinical and imaging features associated with increased mortality in COVID-19 infections. Based on our regression model, advanced age and low oxygen saturation on presentation were independent predictors of mortality, and therefore special attention to these factors may be helpful in the triaging and management of COVID-19 patients.

## Supporting information

S1 Dataset (XLSX)
